# Efficacy and Safety of BCG Revaccination With *M. bovis* BCG Moscow to Prevent COVID-19 Infection in Health Care Workers: A Randomized Phase II Clinical Trial

**DOI:** 10.3389/fimmu.2022.841868

**Published:** 2022-03-22

**Authors:** Laura Raniere Borges dos Anjos, Adeliane Castro da Costa, Amanda da Rocha Oliveira Cardoso, Rafael Alves Guimarães, Roberta Luiza Rodrigues, Kaio Mota Ribeiro, Kellen Christina Malheiros Borges, Ana Carolina de Oliveira Carvalho, Carla Iré Schnier Dias, Aline de Oliveira Rezende, Carine de Castro Souza, Renato Rodney Mota Ferreira, Guylherme Saraiva, Lilia Cristina de Souza Barbosa, Tayro da Silva Vieira, Marcus Barreto Conte, Marcelo Fouad Rabahi, André Kipnis, Ana Paula Junqueira-Kipnis

**Affiliations:** ^1^ Laboratório de Bacteriologia Molecular, Instituto de Patologia Tropical e Saúde Pública, Universidade Federal de Goiás, Goiânia, Brazil; ^2^ Departamento de Biomedicina, Faculdade Estácio de Sá de Goiás, Goiânia, Brazil; ^3^ Faculdade de Medicina, Universidade Federal de Goiás, Goiânia, Brazil; ^4^ Faculdade de Enfermagem, Universidade Federal de Goiás, Goiânia, Brazil; ^5^ Laboratório de Imunopatologia das Doenças Infecciosas, Instituto de Patologia Tropical e Saúde Pública, Universidade Federal de Goiás, Goiânia, Brazil; ^6^ Departamento de Áreas Acadêmicas, Instituto Federal de Goiás, Anápolis, Brazil; ^7^ Programa de Pós-graduação em Ciências da Saúde, Universidade Federal do Maranhão, São Luís, Brazil; ^8^ Departamento de Pesquisa Clínica, Faculdade de Medicina de Petrópolis, Petrópolis, Brazil

**Keywords:** NK cells, innate response, cross protection, respiratory infection, symptoms

## Abstract

The Bacillus Calmette-Guérin (BCG) vaccine, which is widely used to protect children against tuberculosis, can also improve immune response against viral infections. This unicentric, randomized-controlled clinical trial assessed the efficacy and safety of revaccination with BCG Moscow in reducing the positivity and symptoms of COVID-19 in health care workers (HCWs) during the COVID-19 pandemic. HCWs who had negative COVID-19 IgM and IgG and who dedicated at least eight hours per week in facilities that attended to individuals suspected of having COVID-19 were included in the study and were followed for 7, 15, 30, 60, and 180 days by telemedicine. The HCWs were randomly allocated to a revaccinated with BCG group, which received the BCG vaccine, or an unvaccinated group. Revaccination with BCG Moscow was found to be safe, and its efficacy ranged from 30.0% (95.0%CI -78.0 to 72.0%) to 31.0% (95.0%CI -74.0 to 74.0%). *Mycobacterium bovis* BCG Moscow did not induce NK cell activation at 15–20 days post-revaccination. As hypothesized, revaccination with BCG Moscow was associated with a lower incidence of COVID-19 positivity, though the results did not reach statistical significance. Further studies should be carried out to assess whether revaccination with BCG is able to protect HCWs against COVID-19. The protocol of this clinical trial was registered on August 5th, 2020, at REBEC (Registro Brasileiro de Ensaios Clínicos, RBR-4kjqtg - ensaiosclinicos.gov.br/rg/RBR-4kjqtg/1) and the WHO (# U1111-1256-3892). The clinical trial protocol was approved by the Comissão Nacional de ética de pesquisa- CONEP (CAAE 31783720.0.0000.5078).

## Introduction

On March 11, 2020, the World Health Organization (WHO) declared the Coronavirus Disease 2019 (COVID-19), caused by severe acute respiratory syndrome coronavirus 2 (SARS-CoV-2), as a pandemic (1). This disease has caused high morbidity, mortality, and direct and indirect costs for society in general and for countries’ economies, representing a serious public health and socioeconomic problem. Currently, to our knowledge, there is no effective pharmacologic prophylaxis and thus vaccination is the best preventive strategy (2). Despite the rapid advance in the immunization of the world population against COVID-19, the production and acquisition of immunizing agents are still lower than the demand, especially in developing countries like Brazil (3). In addition, new variants (e.g. B.1.1.7 [alpha], B.1.351 [beta], P.1. [gamma], B.1.617.1 [kappa], B.1.617.2 [delta]) and B.1.1.529 [omicron] of SARS-CoV-2 have emerged that may increase the pathogenic potential of the virus (4, 5).

The Bacillus Calmette-Guérin (BCG) vaccine has been safely and widely used in newborns and children for 100 years. Composed of attenuated *Mycobacterium bovis*, this vaccine prevents disseminated childhood tuberculosis (TB) and meningitis (6). Previous epidemiological studies have shown that BCG vaccination was associated to reduced child mortality from all causes (7, 8). Other studies proved that the vaccination with BCG could confer immunity against viral respiratory tract infections in children (9). Later, immunological studies demonstrated that the administration of the BCG vaccine provided a non-specific immunomodulatory effect, called trained immunity, that was associated with cross-protection against other infections (10). Trained immunity is mediated by the epigenetic, transcriptional, and functional reprogramming of innate immune cells (monocytes, macrophages, and natural killer [NK] cells), potentially increasing the production of cytokines (10, 11). Shortly after, a meta-analysis of three trials reinforced that early administration of BCG in low weight infants was associated with major reductions in mortality rate (12). It has recently been demonstrated that the vaccination with BCG can confer immunity against viral respiratory tract infections in elderly (13, 14).

The mild manifestations of COVID-19 among children, seen in ecological studies performed in the early stages of the pandemic, were associated with trained immunity generated using vaccines such as BCG (15). However, these studies were heavily criticized for not systematically correcting confounding variables such as socioeconomic differences, demographic structure, time of arrival of the COVID-19 pandemic, population comorbidities, testing capacity, and control strategies between countries (16, 17). To our knowledge, studies that evaluated BCG vaccine efficacy against COVID-19 in health care workers (HCWs) are scarce or are still ongoing. Thus, this prompted us to design a randomized clinical trial to evaluate the efficacy and safety of BCG revaccination among HCWs at a high risk for COVID-19 infection to prevent disease or decrease symptoms and to improve innate immune response.

## Methods

### Trial Design

This study was a unicentric, parallel, randomized, phase II clinical trial conducted among HCWs with no prior COVID-19 infection. The study was conducted at the Federal University of Goiás (UFG), Brazil, between August 20^th^, 2020, and August 31^st^, 2021. The protocol of this clinical trial was registered on August 5^th^, 2020, at REBEC (Registro Brasileiro de Ensaios Clínicos, RBR-4kjqtg- ensaiosclinicos.gov.br/rg/RBR-4kjqtg/1) and the WHO (# U1111-1256-3892). The clinical trial protocol was approved by the Comissão Nacional de ética em pesquisa- CONEP (CAAE 31783720.0.0000.5078). The detailed study protocol was published as appendix data in a previous publication (18). This study complied with guidelines outlined under the Consolidated Standards of Reporting Trials (CONSORT) (19).

### Participants

The HCWs were recruited at Hospital das Clínicas (HC/UFG) or Hospital Estadual Geral de Goiânia Dr. Alberto Rassi (HGG), or by independently completing an online recruitment form. Inclusion and exclusion criteria were described previously (18) and are described next. Inclusion criteria: Eligible individuals were HCWs working at least eight hours per week in a medical facility attending to confirmed or suspected COVID-19 patients, aged 18 years or older, with a history of previous BCG vaccination and with no history of COVID-19. Potential study volunteers underwent a screening evaluation that included an explanation of the study and an invitation to take part in it. After signing the informed consent form, sociodemographics, comorbidities, lifestyle habits, use of medications, information about contacts with people with COVID-19 were collected. In addition, all HCWs were asked about their previous history of vaccination with BCG, and the region of the deltoid muscle on the upper external surface of the right arm was observed to verify the presence of a BCG vaccine scar. Exclusion criteria: Subjects with prior known reaction to the BCG vaccine, fever in the previous 24 hours, pregnant or breastfeeding women, suspected or confirmed viral infection including COVID-19 or bacterial infection, previous diagnosis of tuberculosis, vaccination in the previous four weeks, medical diagnosis of immunosuppressive diseases, such as human immunodeficiency virus (HIV), and/or cancer in the previous two years and/or autoimmune disease and/or use of corticosteroids and/or antibiotics and/or chemotherapy were not eligible to participate in the study. Also, at enrollment, subjects with positive IgM and/or IgG for COVID-19 and/or neutrophil counts bellow 500/mm^3^ were not enrolled in the study. Participants were randomized to one of two arms: a revaccination with *M. bovis* BCG Moscow arm or an unvaccinated arm (no placebo vaccine was used). This study was conducted in accordance with the requirements for Good Clinical Practice ICH E6 (R2).

Blood samples from all included HCWs were processed and serum, plasma, PBMCs, and total blood were stored for immunological assays. Initially, enrolled subjects were subsequently excluded if they received a COVID-19-positive test on day 15 after randomization. However, the study protocol was modified and participants who tested positive for COVID-19 within the first 15 days after randomization were kept in the study. This alteration was approved by the Comissão Nacional de Ética em Pesquisa- CONEP and did not affect the integrity of the interpretation of the results obtained and allowed for the assessment of the maximum accurate description of the efficacy of the BCG vaccine since its administration.

### Definitions, Intervention, and Follow-Up

Flu-like syndrome symptoms according to the recommendations of the Brazilian Ministry of Health were defined as: dry or productive cough, hemoptysis, fever, sore throat, night sweats, shortness of breath, reduced sense of smell or taste, lack of appetite, diarrhea, headache, rhinorrhea, nasal congestion, asthenia/fatigue, and/or myalgia (20).

COVID-19 was considered for an individual presenting an IgM/IgG test or RT-PCR positive test. Any participant that presented symptoms related to flu-like syndrome or symptoms associated to COVID-19 were oriented to perform an RT-PCR test. The severity of COVID-19 was classified according to the WHO guidelines (21) as follows. (1) Asymptomatic: without any symptoms or signs, but with a laboratory diagnosis; (2) Mild/moderate condition: dry cough, fever (regardless of temperature), headache, mild difficulty in breathing defined by a respiratory rate up to 30 breaths per minute and oxygen saturation measured by digital oximetry that was greater than or equal to 90% in ambient air; (3) Severe: signs of pneumonia (fever, cough, dyspnea, tachypnea) and one of the following: respiratory rate > 30 bpm; acute respiratory distress syndrome; SaO2 < 90% in ambient air; (4) Critical condition: admission to the ICU, need for ventilatory support (NIV, HNFA, IMV), use of vasoactive drugs.

Enrolled subjects either received or did not receive a single dose of BCG Moscow vaccine *via* intradermal administration. All participants were followed and evaluated through telemedicine on days 7, 15, 30, 60, and 180. An assessment tool designed for this study to evaluate signs of flu-like syndrome symptoms (20) and possible adverse events (AEs) was used (22). Also, participants were able to contact the medical team, if needed, to report a clinical sign and/or symptom and/or confirmation of COVID-19 diagnosis and/or for medical assistance. Participants suspected of contamination with SARS-CoV-2 were invited to submit to serological and/or molecular tests and clinical evaluation. Participants diagnosed with COVID-19 were referred for treatment to accredited health facilities for the care of COVID-19 cases. Fifteen days after randomization, a new blood sample was collected from the randomized HCWs for COVID-19 testing and immune response evaluation. After 180 days of inclusion, all HCWs were contacted for final blood collection for COVID-19 testing. At the end of the study, the results of the BCG-vaccinated and unvaccinated groups were unblinded and compared according to primary and secondary outcomes.

### Interventions After Initiation of Specific Vaccination Against COVID-19

Although not envisioned in the trial design, specific vaccines against COVID-19 were approved during the trial in Brazil and were made available for the vaccination of priority groups, which included HCWs. To avoid any bias in data interpretation, all HCWs included in this clinical trial who did not develop symptomatic COVID-19 were invited to submit to a third blood draw for COVID-19 serological testing before their COVID-19-specific vaccination or within 14 days thereafter. This additional information as well as the COVID-19 vaccine types was recorded, and these participants were followed by telemedicine for the diagnosis of COVID-19 for 180 days.

### Outcomes

Primary: Reduction of positivity for COVID-19 through serological and molecular tests and clinical evaluation among individuals vaccinated with BCG. Reduction of COVID-19 symptoms among HCWs revaccinated with BCG was verified by telemedicine and clinical evaluation during follow-up for 180 days.

Secondary: Innate immune activation among individuals in the BCG-vaccinated group was verified by NK cell population activation analysis after 15–20 days post-randomization compared to day 1 (the day of inclusion in the study).

### Safety Evaluation

Adverse events (AEs) and serious adverse events (SAEs) were reported from baseline until the last patient’s evaluation for all included participants. SAEs and all other AEs were reported according to NIH and FDA guidelines (23, 24). An adverse event was defined as any reaction that was not present before the start of the study (exposure to study vaccination) or any pre-existing reaction that worsened in intensity and frequency after exposure. In this study, SAEs were defined as events that were life-threatening, resulted in initial or prolonged hospitalization, caused irreversible, persistent, or significant disability and/or incapacity, required intervention to prevent harm, or had other medically serious consequences. All other AEs were reported as non-severe. All AEs were graded as grade I (transient and well-tolerated by the patient), grade II (causing discomfort and causing disability), grade III (affecting the usual activities to an important degree and causing disability) while SAEs were classified as grade IV, indicating a potentially life-threatening event.

BCG vaccine-related AEs (adverse reactions) were questioned to all trial participants. As this study did not use a placebo vaccine, only participants allocated to the BCG-vaccinated group reported any vaccine-related AE. Therefore, vaccine-related AEs were measured in analysis only for the intervention group. According to the Brazilian Ministry of Health (22), severe local AEs related to BCG vaccination (severe adverse reactions) are local abscess keloid, cutaneous skin lesions, and lymphadenitis suppuration, while possible severe systemic AEs related to BCG vaccination are osteitis, disseminated BCG, and immune reconstitution syndrome.

### Randomization

Randomization of 400 consecutive numbers was performed on the online platform Randomization.com (http://www.jerrydallal.com/random/permute.htm) in blocks of 20 (see **Supplementary Materials** for detailed information). In order to randomly allocate recruited individuals to the vaccination or unvaccinated groups and, at the same time, optimize the use of the BCG vaccine, which could not be stored for more than six hours after the vaccine vial was opened. 400 consecutive numbers ranging from BCG001 to BCG400 were inserted on the online platform Randomization.com (http://www.jerrydallal.com/random/permute.htm) with the permutable treatment labels “BCG vaccination” and “No vaccination” to designate whether the individual would be vaccinated or not, respectively. The total number of subjects was set to 400, and the randomization was divided into blocks of 20 with 20 subjects each. A collaborator who was not involved with the recruitment manipulated the randomization output. Four hundred envelopes externally labeled with the consecutive numbers BCG001 to BCG400 received the respective randomization output printed on a piece of paper with the same BCG number and the result “BCG vaccination” or “No vaccination” generated by the computer software. All 400 envelopes were sealed and ready as of day 1 of the subjects’ recruitment. On the day of recruitment, a consecutive BCG number was assigned to each individual included in the study and a sealed envelope with the exact same BCG number was opened in front of the participant, at which point both the participant and the study staff learned whether the individual was allocated to the vaccination group or not. If allocated to the vaccination group, the individual was transported to the vaccination site; if not, the individual was informed once more about the importance of the unvaccinated group for the trial and their contribution in continuing to participate in the project until the end.

At the vaccination site, a new vial of BCG Moscow was suspended with 1 mL of the accompanying diluent and maintained at 4–8°C in a cooler until use. For each participant assigned to be vaccinated, a nurse with ample experience with BCG vaccination retrieved 100 μL of the vaccine immediately prior to vaccination and injected the whole volume intradermally in the region of the upper right arm. The individual received a flyer with possible AEs of the BCG vaccine and a physician contact telephone number in case of any additional concerns. Inquiries about AEs related to BCG vaccination began at seven days post-randomization or were spontaneously reported if the participant contacted the physician with any concern regarding the vaccination.

### Blinding

The study was blinded to laboratory researchers, to those who evaluated the results, and to those who performed the statistical analyses. In this case, only the participant’s identification number was made available. There was no blinding for health professionals participating in this trial. The statistician first had access to data coded only by the participant’s ID, and only after the conclusion of the study and approval by the Data and Safety Monitoring Board were the data unmasked and analyzed accordingly. The data remained blinded to the researchers.

### Innate Immune Response After BCG Vaccination

Heparinized total blood was withdrawn from the participants on the day of inclusion and 15–20 days after randomization. Five hundred microliters of blood were aliquoted in cryogenic tubes containing 500 μL of freezing solution composed of 20% DMSO and 80% bovine serum albumin (BSA). All blood samples were kept at -80°C until their cell preparations for cytometry. Using a 37°C water bath, the cells were quickly thawed and distributed in 50 mL tubes or 48-well plates. NK cell staining was performed according to a protocol standardized in our laboratory. In detail, on the day of the cell analyses, two samples of blood from each participant were processed, one collected on the day of recruitment, and one collected at 15–20 days post-randomization. To evaluate NK cell activation, 500 μL of the cell suspensions were transferred to 50 mL conical tubes and erythrocytes were lysed with lysis buffer (0.15 M NH4Cl, 10 mM KHCO3). After washing the cells with saline, the cell pellet was resuspended with 800 μL of complete RPMI (cRPMI; GIBCO, Invitrogen Corporation Grand Island, NY, USA) containing glutamine (200 mM; Sigma–Aldrich-Brazil, São Paulo), pyruvate (10 nM; Sigma–Aldrich-Brazil, São Paulo), non-essential amino acids (2 mM; Sigma–Aldrich-Brazil, São Paulo), 50 μg/mL of penicillin/streptomycin (1.000 U/mL GIBCO) and 10% BSA. The cells were counted and adjusted to 10^6^ cells/mL and distributed in 96-well culture plates (200 μL/well). Cells were stimulated with medium or with culture filtrate proteins (CFPs) from BCG (0.5 μg/μL) after resting for two hours at 37°C in a 5% CO_2_ incubator. Then, the cells were incubated for 17 hours, and monensin (3 mM; eBioscience) was added to the cultures, which were further incubated for 5 hours. After this period, the plates were centrifuged at 2000 xg for 15 minutes at 10°C and the cells were resuspended and treated with 20 μL of mouse sera to block FC receptors. After 10 minutes of incubation at 4–8°C, the cells were incubated for 30 minutes at the same temperature with monoclonal antibodies against surface markers diluted according to a previous standardization. According to the analysis that was to be performed, a combination of the following antibodies were used: mouse anti-human CD16-FITC, CD314-PerCP-eFluor™ 710, TCR-PE-Cyanine7, CD57-eFluor 660, CD27-APC, CD3-Alexa Fluor 700, and CD56 APC-eFluor 780 (all antibodies were from eBioscience; the clones of all antibodies used in this study are presented in **Table 1**). Next, the cells were incubated for 20 minutes with Perm Fix (100 μL), followed by the addition of 100 μL of Perm wash for a further 20 minutes. After centrifuging the plates at the established conditions, the antibodies against intracellular cytokines were added diluted in Perm wash solution (BD Pharmingen, San Jose, CA, EUA) and the plates were incubated for 30 minutes at 4–8°C. For this purpose, mouse anti-human IFN-γ-PE and TNF-α-PE were used. Then, the plates were centrifuged, and the pellet was resuspended in 400 μL of 0.05% sodium azide PBS containing 0.05% sodium azide. The paired samples corresponding to day 1 and 15 for each participant were always processed and analyzed concomitantly. The cells were immediately acquired using Attune™ NxT (ThermoFisher). To evaluate NK cells, at least 50,000 events were acquired and analyzed. The data were evaluated using FlowJo Version 7.0 software (FlowJo™). All cells were gated to exclude doublets using FCS-A and FCS-H parameters. After this, using granularity and size, lymphocytes were gated and cells CD16^+^CD56^+^ were selected. These cells were analyzed for CD3 expression, and cells CD16^+^CD56^+^ CD3^-^ were considered NK cells.

**Table 1:** Antibodies used in this study (all from eBioscience™).

Fluorochrome	Marker	Catalog #	Channel	Clone
FITC	CD16	11-0168-42	BL1	eBioCB16 (CB16)
PE	CD49d	12-0499-42	BL-2	9F10
PE	IFN-γ	12-7319-42	BL-2	4S.B3
PE	TNF-α	12-7349-82	BL-2	MAb11
PercP	CD63	MA110269	BL-3	MEM-259
PerCP-eFluor™ 710	CD314 (NKG2D)	46-5878-42	BL-3	1D11
PE-Cyanine7	CD15	25-0159-42	BL-4	HI98
PE-Cyanine7	TCR V alpha 24 J alpha 18	25-5806-42	BL4	6B11
eFluor^®^ 660	CD57	50-0577-42	RL-1	TB01 (TBO1)
APC	CD27	17-0279-42	RL-1	O323
APC	LAP	17-9829-42	RL-1	FNLAP
APC	CD66	17-0668-42	RL-1	CD66a-B1.1
Alexa Fluor 700	CD3	56-0038-42	RL-2	UCHT1
Alexa Fluor 700	ARGINASE	56-3697-82	RL-2	A1exF5
Alexa Fluor 700	CD14	56-0149-42	RL-2	61D3
APC-eFluor^®^ 780	CD56 (NCAM)	47-0452-82	RL-3	RA3-6B2
APC-eFluor^®^ 780	CD123	47-1239-42	RL-3	6H6
APC-eFluor^®^ 780	CD16	47-0168-42	RL-3	eBioCB16 (CB16)

LAP, latency-associated peptide.

### Sample Size

The sample size calculation, which was already published (18), was performed according to the formula outlined by Hulley et al. (25). Thus, 197 HCWs would be allocated to each intervention group. As mentioned above, with the initiation of COVID-19-specific vaccination of HCWs, the study was unmasked in September 2021, before reaching the target numbers but without compromising the analysis, since most of HCWs became vaccinated against COVID19. This, a new sample size calculation was performed based on new publication of COVID-19 which evaluated the effectiveness of BCG revaccination in individuals at risk (26). Thus, considering a statistical power of 80%, significance level of 5%, equal allocation rate between groups and a proportion of the primary outcome (development of COVID-19) in the control group of 51.5% and in the intervention group of 25.9%, effect size of 50.0%, drop rate of 68% and clinically acceptable margin of 0.15, the minimum necessary sample size was estimated at 104 patients (52 in the unvaccinated group and 52 in the intervention group).

### Statistical Methods

#### Baseline Comparison

The unvaccinated and BCG groups had their demographic, clinical, and behavioral characteristics analyzed and compared. Pearson’s chi-squared test with Yates’ continuity correction or Fisher’s exact tests were used to compare the proportions of categorical variables. A Student’s t-test (normal distribution) or a Mann-Whitney U test with continuity correction (non-normal distribution) was used to compare the means or distributions of continuous variables. The analysis of the normality of continuous variables was performed using the Kolmogorov-Smirnov test with Lilliefors correction. Parametric quantitative variables were evaluated using a paired t-test or a Wilcoxon test; for non-parametric quantitative variables.

#### Vaccine Efficacy (VE)

Population analysis was performed to estimate VE in the prevention of COVID-19. VE was calculated by two statistical methods: a Poisson regression model and a Cox proportional regression model. A Poisson regression model was used to estimate the incidence rate ratio (IRR) (27). The terms included in the model (explanatory variables) were the study group (BCG group versus control group), age at randomization, and sex. Even with a balance between groups, age was included in the model due to its potential influence on the adjusted measures in VE studies; sex was also included due to a *P-*value <0.10 observed in group comparisons. Also, the log of the risk period to the primary outcome was used as the model’s offset variable to adjust for participants with different study follow-up times. A Cox proportional regression model was used to estimate the hazard ratio (HR). The terms included in this model (explanatory variables) were the study group (BCG group versus control group), age at randomization, and sex. VE was defined as the percentage reduction in the IRR or HR for the primary outcome and was calculated as 1-aIRR (Poisson model) or 1-HR (Cox model), accompanied by the respective 95% CI. Cumulative incidences of cases were presented using the Kaplan-Meier method.

The first vaccine against COVID-19 was approved for use in Brazil in January 2021, during the follow-up of participants in this clinical trial. Thus, two analyses were performed for VE at endpoints. In the first scenario, entitled “without censoring for COVID-19 vaccination”, we included all cases of COVID-19 until the end of the follow-up, regardless of the participant’s COVID-19 vaccination status. In this case, the time of the event was from the date of inclusion to the date of development of the primary outcome; the participant was censored when (i) follow-up was missed or (ii) 180 days of follow-up had been completed. In the second scenario, entitled “with censoring for COVID-19 vaccination”, individuals were censored from the analysis after 14 days of the subject’s first dose of COVID-19 vaccination, independent of the producing laboratory. In this scenario, the participant was censored when (i) the follow-up was missed (ii) after 14 days of the first vaccine dose (if the participant received the COVID-19 vaccine), or (iii) 180 days of follow-up had been completed (if the participant did not receive the COVID-19 vaccine). Similarly, vaccine efficacies were evaluated according to symptomatic COVID-19 cases.

### Registration and Protocol

The protocol was registered on August 5th, 2020, at REBEC (Registro Brasileiro de Ensaios Clínicos, RBR-4kjqtg) and the WHO (# U1111-1256-3892). The clinical trial protocol was approved by the Brazilian Ethics Committee (CAAE 31783720.0.0000.5078).

This study complied with guidelines outlined under the Consolidated Standards of Reporting Trials (CONSORT).

## Results

### Recruitment and Participants

**Figure 1:**
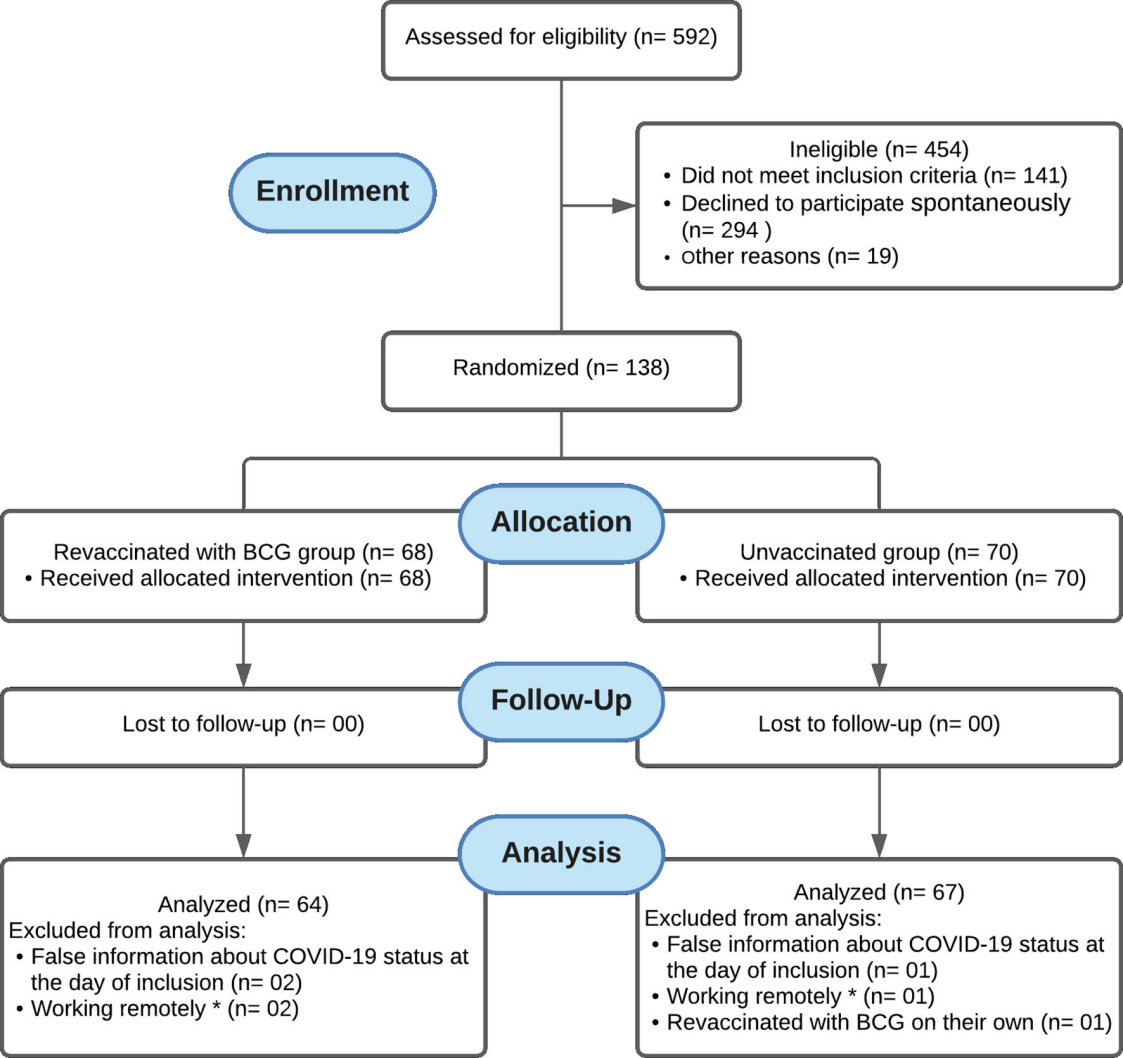
Flow diagram showing that 592 HCWs were recruited, of whom 454 were ineligible (141 did not meet the inclusion criteria, 294 declined to participate, and 19 were excluded for other reasons) and 138 were randomized. Of the randomized HCWs, 68 were allocated to the revaccinated with BCG group and 70 to the unvaccinated group. There was no loss to follow-up in neither group. In the revaccinated BCG group, two participants were excluded from analysis due to false information about COVID-19 status on the day of inclusion, and another two were excluded for working remotely, remaining 64 participants for analysis. In the unvaccinated group, one participant was excluded from analysis due to false information about COVID-19 status on the day of inclusion, another one was excluded for working remotely, and one was excluded for having been vaccinated with BCG on their own, leaving 67 participants for analysis. * Health care workers in remote jobs refers to HCWs who were not working for at least eight hours per week under exposure to those suspected of being infected with COVID-19.

This clinical trial recruited 592 HCWs, of whom 454 were ineligible (141 for not meeting inclusion criteria, 294 for declining to participate spontaneously, and 19 for other reasons) and 138 were randomized, as summarized in **Figure 1**. Of the randomized HCWs, 68 were allocated to the revaccinated with BCG group and 70 to the unvaccinated group. In the revaccinated-BCG group, two participants were excluded from analysis due to false information about COVID-19 status on the day of inclusion, and two participants were excluded from analysis because they were working remotely. Thus, 64 participants were considered for analysis. In the group unvaccinated with BCG, one participant was excluded from analysis due to false information about COVID-19 status on the day of inclusion, one participant was excluded from analysis because he was working remotely, and one participant revaccinated with BCG on their own. Thus, 67 participants were considered for analysis (**Figure 1**). The sample consisted of HCWs categorized as administrative staff (n = 13; 9.9%), nurse staff (n = 48; 36.6%), dental professionals (n = 9; 6.9%), laboratory staff (n = 12; 9.2%), nutritionists (n = 4; 3.1%), community health agents (n = 6; 4.6%), paramedics (nurses) (n = 18; 13.7%), medical staff (n = 10; 7.6%), and other HCWs (n = 11; 8.4%).

The baseline characteristics of participants according to allocated group (unvaccinated and revaccinated with BCG) are shown in **Table 2**. The results indicated that all sociodemographic and clinical variables, use of medications, and baseline laboratory values were balanced between the unvaccinated and revaccinated with BCG groups, with no statistically significant difference (*P* > 0.05). In addition, all HCWs included in the study reported a previous history of BCG vaccination and there was no statistically significant difference between the percentage of individuals with and without a scar from previous BCG vaccination between the BCG-revaccinated and unvaccinated groups.

**Table 2:** Baseline characteristics according to allocated group (Unvaccinated or revaccinated with BCG).

Variables	All (n = 131)	Unvaccinated (n = 67)	Revaccinated with BCG (n = 64)	*P* -value
Age (years), mean (SD)	43.0 (11.2)	44.2 (11.3)	41.8 (11.0)	0.23*
Age distribution (years), n (%)				
18-59	119 (90.8)	60 (89.6)	59 (92.2)	0.60^†^
≥ 60	12 (9.2)	7 (10.4)	5 (7.8)	
Sex, n (%)				
Female	100 (76.3)	56 (83.6)	44 (68.8)	0.07^†^
Male	31 (23.7)	11 (16.4)	20 (31.3)	
Economic class, n (%)^||^				
A or B	37 (28.2)	20 (29.9)	17 (27.0)	0.79^†^
C	62 (47.7)	30 (44.8)	32 (50.8)	
D or E	31 (23.8)	17 (25.4)	14 (22.2)	
Number of contacts with suspected COVID-19 patients, median (IQR)	20.0 (8.0-50.0)	20.0 (7.5-50.0)	17.5 (8.0-40.0)	0.78^§^
BMI (Kg/m^2^), mean (SD)	27.0 (5.0)	27.3 (4.9)	26.7 (5.0)	0.49*
Obesity, n (%)	31 (23.7)	18 (26.9)	13 (20.3)	0.50^†^
Comorbidity, n (%)	25 (19.1)	14 (20.9)	11 (17.2)	0.75^†^
Comorbidities, n (%)				
Hypertension	19 (14.5)	10 (14.9)	9 (14.1)	1.00^†^
Diabetes	4 (3.1)	4 (2.0)	0	0.12^‡^
Cardiac insufficiency	4 (3.1)	2 (3.0)	2 (3.1)	1.00^‡^
Renal insufficiency	0	0	0	N/A
Current smoking, n (%)	3 (2.3)	1 (1.5)	2 (3.1)	0.61^‡^
Alcohol addiction, n (%)	8 (6.1)	3 (4.5)	5 (7.8)	0.49^‡^
BCG vaccine scar*, n (%)	113 (86.3)	57 (85.1)	56 (87.5)	0.88^†^
Medication use, n (%)				
Antihypertensive	19 (14.5)	11 (16.4)	8 (12.5)	0.70^†^
Antiarrhythmic	4 (3.1)	2 (3.0)	2 (3.1)	1.00^‡^
Insulin	1 (0.8)	1 (1.5)	0	1.00^‡^
Antibiotic	0	0	0	N/A
Anticoagulant	0	0	0	N/A
Vitamin supplement	43 (32.8)	24 (35.8)	19 (29.7)	0.58^†^
Ivermectin	4 (3.1)	2 (3.0)	2 (3.1)	1.00^‡^
Hydroxychloroquine	0	0	0	N/A
Hematological parameters				
Red cells (tera/L), median (IQR)	4.7 (4.4-5.0)	4.7 (4.4-5.0)	4.8 (4.4-5.1)	0.46^§^
Hematocrit (%), median (IQR)	42.7 (41.9-45.6)	42.6 (40.8-45.6)	43.1 (41.3-45.2)	0.46^§^
Hemoglobin (g/dL), mean (SD)	14.1 (1.5)	14.1 (1.4)	14.2 (1.5)	0.58*
MCV (IL), median (IQR)	91.2 (88.6-93.6)	91.4 (88.9-94.6)	91.4 (88.6-93.1)	0.50^§^
MCH, median (IQR)	29.7 (28.7-30.8)	29.7 (28.8-31.0)	29.6 (28.4-30.5)	0.58^§^
CHCM (g/dL), mean (SD)	32.6 (0.9)	32.6 (1.0)	32.6 (0.9)	0.83*
RDW (%), median (IQR)	12.2 (11.8-12.7)	12.2 (11.8-12.7)	12.3 (11.9-12.8)	0.50^§^
WBC (x10^9^/L), mean (SD)	6.1 (1.5)	6.0 (1.4)	6.2 (1.7)	0.51*
Neutrophils (x10^9^/L), median (IQR)	3.2 (2.6-4.0)	3.3 (2.7-3.9)	3.2 (2.5-4.2)	1.00^§^
Lymphocytes (x10^9^/L), mean (SD)	2.2 (0.7)	2.2 (0.6)	2.3 (0.8)	0.25*
Monocytes (x10^9^/L), median (IQR)	0.3 (0.2-0.4)	0.3 (0.2-0.4)	0.3 (0.2-0.4)	0.38^§^
Platelets (x10^9^/L), median (IQR)	245.0 (209.5-283.0)	250.0 (215.5-286.5)	244.0 (204.0-280.5)	0.64^§^

SD, standard deviation; BCG, Bacilli de Calmette and Guérin; COVID-19, coronavirus disease 2019; IQR, interquartile range; BMI, body mass index; MCH, mean corpuscular hemoglobin; MCV, mean corpuscular volume; CHCM, mean corpuscular hemoglobin concentration; N/A, not applicable; RDW, red cell distribution width; WBC, white blood cells; *Student’s t-test for independent samples; ^†^Pearson’s chi-squared test with Yate’ continuity correction; ^‡^Fisher’s exact test; ^§^Mann-Whitney U test with continuity correction; ^||^Missing data=1. *The BCG vaccine scar was verified at the time of inclusion of the HCW in the study.

### Vaccine Efficacy to Prevent COVID-19 Infection and Symptomatic COVID-19

**Figure 2:**
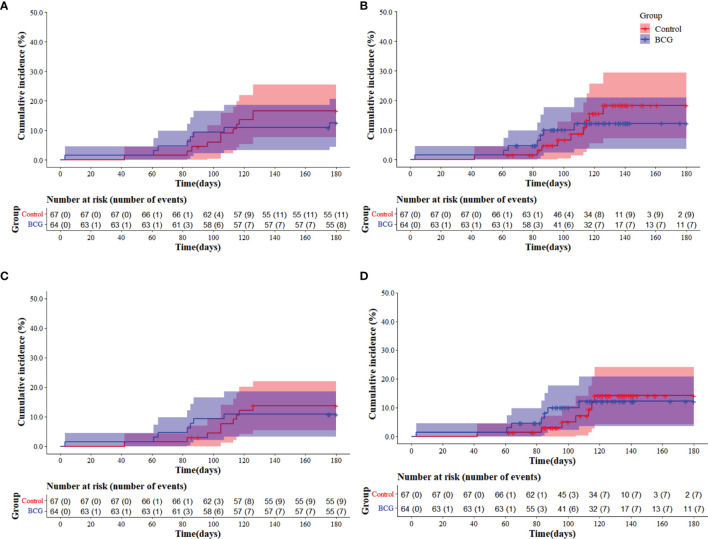
Cumulative incidence Kaplan-Meier curves of COVID-19 cases (laboratory-confirmed COVID-19) in two scenarios: without censoring for COVID-19 vaccination **(A)** and with censoring for COVID-19 vaccination **(B)**. The cumulative incidence Kaplan-Meier curves of symptomatic COVID-19 cases are also shown in two scenarios: without censoring for COVID-19 vaccination **(C)** and with censoring for COVID-19 vaccination **(D)**. Blue lines and shadings represent the revaccinated with BCG group (BCG) cumulative incidence of cases and 95% CI, respectively. Red lines and shadings represent the unvaccinated group (Control, unvaccinated) cumulative incidence of cases and 95% CI, respectively.

As detailed in methods, during the trial period, specific COVID-19 vaccines became available to HCWs, thus the VE is presented in two scenarios (**Figure 2**; **Table 3**). The Kaplan-Meier cumulative incidence curves of the primary endpoint for the scenarios without censoring for COVID-19 vaccination (**Figure 2A**) and with censoring for COVID-19 vaccination (**Figure 2B**) are shown in **Figure 2**. Although the COVID-19 cumulative cases were smaller in the revaccinated with BCG group than in the unvaccinated group, no differences were observed between the curves (**Figure 2A**). Similarly, considering censoring for COVID-19 vaccination, no differences were observed between the cumulative incidence curves (**Figure 2B**). The general VE based on the Cox proportional model was 30.0% (95.0% CI: -78.0 to 72.0%) and, based on the Poisson model, 31.0% (95.0% CI: -74.0 to 74.0%) (**Table 3**). In this scenario (n = 115. 87.8%), nine COVID-19 cases were detected in the unvaccinated group and seven in the revaccinated with BCG group. Thus, the VE based on the Cox proportional model in this scenario was 26.0% (95.0% CI: -107.0 to 73.0%) and, based on the Poisson model, 32.0% (95.0% CI: -89.0 to 77.0%) (**Table 3**).

**Table 3:** Vaccine efficacy based on COVID-19 infection in both scenarios: without censoring for COVID-19 vaccination and with censoring for COVID-19 vaccination.

Scenario	All	Unvaccinated	Revaccinated with BCG
**COVID-19 –** without censoring for COVID-19 vaccination			
Number of COVID-19 cases, n	19	11	8
Cumulative incidence, n/total (%)	19/131 (14.5)	11/67 (16.4)	8/64 (12.5)
Number censored, n (%)	112 (85.5)	56 (83.6)	56 (87.5)
VE based on HR (95.0% CI)*			30.0 (-78.0 to 72.0)
Person-Years	59.8	30.4	29.4
IR per 100 Person-Years (95.0% CI)	31.8 (19.1-49.6)	36.2 (18.1-64.7)	27.2 (11.7-53.6)
**VE based on IRR (95.0% CI) ^†^**			31.0 (-74.0 to 74.0)
**COVID-19 –** with censoring for COVID-19 vaccination			
Number of COVID-19 cases, n	16	9	7
Cumulative incidence, n/total (%)	16/131 (12.2)	9/67 (13.4)	7/64 (10.9)
Number censored, n (%)	115 (87.8)	58 (86.6)	57 (89.1)
VE based on HR (95.0% CI)*			26.0 (-107.0 to 73.0)
Person-Years	42.2	21.2	21.0
IR per 100 Person-Years (95.0% CI)	37.9 (21.7-61.6)	42.5 (19.4-80.6)	33.3 (13.4-68.7)
**VE based on IRR (95.0% CI)^†^**			32.0 (-89.0 to 77.0)

The primary endpoint was COVID-19 defined as the presence of positivity by RT-PCR or IGM or IgG serology defined as presented in methods.

IRR, incidence rate ratio; HR=hazard ratio; VE, vaccine efficacy; CI, confidence interval. *Vaccine efficacy based on 1-HR obtained in Cox proportional model adjusted for age and sex. ^†^Vaccine efficacy based on 1-IRR obtained in Poisson model adjusted for age and sex, with the natural logarithm (log[n]) of time at risk as offset variable.

The Kaplan-Meier cumulative incidences of symptomatic COVID-19 were evaluated for the scenarios without censoring for COVID-19 vaccination (**Figure 2C**) and with censoring for COVID-19 vaccination (**Figure 2D**). No differences were observed between the curves in both scenarios. The VE result for this outcome based on the Cox proportional model was 28.0% (95.0% CI: -98.0 to 74.0%) and, based on the Poisson model, 30.0% (95.0% CI: -93.0 to 76.0%), since nine cases of COVID-19 were observed in the unvaccinated group and seven in the revaccinated with BCG group (**Table 4**). In the scenario with COVID-19-specific vaccination, the VE for symptomatic COVID-19 was 8.0% (95.0% CI: -173.0 to 69.0%) based on the Cox proportional model and based on the Poisson model, 16.0% (95.0% CI: -155.0 to 72.0%) (**Table 4**).

**Table 4:** Vaccine efficacy based on symptomatic COVID-19 infection in both scenarios: without censoring for COVID-19 vaccination and with censoring for COVID-19 vaccination.

Scenario	All	Unvaccinated	Revaccinated with BCG
**COVID-19 –** without censoring for COVID-19 vaccination			
Number of COVID-19 cases, n	16	9	7
Cumulative incidence. n/total (%)	16/131 (12.2)	9/67 (13.4)	7/64 (10.9)
Number censored, n (%)	115 (87.8)	58 (86.6)	57 (89.1)
VE based on HR (95.0% CI)^*^			28.0 (-98.0 to 74.0)
Person-Years	59.8	30.4	29.4
IR per 100 Person-Years (95.0% CI)	26.8 (15.3-43.4)	29.6 (13.5-56.2)	23.8 (9.6-49.1)
**VE based on IRR (95.0% CI)^†^**			30.0 (-93.0 to 76.0)
**COVID-19 –** with censoring for COVID-19 vaccination			
Number of COVID-19 cases, n	14	7	7
Cumulative incidence, n/total (%)	14/131 (10.7)	7/67 (10.4)	7/64 (10.9)
Number censored, n (%)	117 (89.3)	60 (89.6)	57 (89.1)
VE based on HR (95.0% CI)*			8.0 (-173.0 to 69.0)
Person-Years	42.2	21.2	21.0
IR per 100 Person-Years (95.0% CI)	33.2 (18.1-55.7)	33.0 (13.3-68.0)	33.3 (13.4-68.7)
**VE based on IRR (95.0% CI)^†^**			16.0 (-155.0 to 72.0)

Symptomatic COVID-19 defined as the presence of RT-PCR or IgM or IgG serology positive test and presence of flu-like symptoms. IRR, incidence rate ratio; HR=hazard ratio; VE, vaccine efficacy; CI, confidence interval. ^*^Vaccine efficacy based on 1-HR obtained in Cox proportional model adjusted for age and sex; ^†^Vaccine efficacy based on 1-IRR obtained in Poisson model adjusted for age and sex, with the natural logarithm (log[n]) of time at risk as offset variable.

Additionally, the efficacies calculated considering only HCWs that presented previous BCG scar showed no significant differences between the groups that were revaccinated with BCG or unvaccinated (VE varied from 20.0 to 24.0; **Table S1**). In this study, 31 men were included (11 in the unvaccinated group and 20 in the revaccinated group). Five COVID-19 positive cases were seen among male HCWs, most of them occurred among individuals revaccinated with BCG (4/20; 20%). One hundred females were enrolled and evaluated, among them, 14 COVID-19 positives were observed. Most of the positive cases happened among HCW females unvaccinated with BCG (10/56; 17.9%; **Table S2**). In female HCWs, VE based on Cox analysis was 52.0% (95.0% CI: -52.0 to 85.0%). While our data showed that females were protected for COVID-19 and males presented a negative VE (-132.0, 95% CI: -2,040.0-75.0), the analysis did not show statistically significant difference (**Table S2**).

Comparing the symptoms presented by HCWs that had symptomatic COVID-19, no significant differences between the revaccinated with BCG group and the unvaccinated group was observed (**Table S3**). For this analysis purposes, the signs and symptoms reported were grouped into: (i) systemic: night sweats, fever, myalgia, fatigue and, arthralgia;(ii) high respiratory: sore throat, runny nose and, nasal congestion; (iii) low respiratory: cough and dyspnea; (iv) gastrointestinal: nausea, vomiting, and diarrhea; (v) neurological: headache, anosmia, and ageusia; (vi) flu syndrome.

The comparison of general symptoms reported by HCWs included in the unvaccinated and revaccinated with BCG groups is described in **Table S4**. The results indicated that clinical symptoms were similar between the HCWs included in BCG and unvaccinated groups during the follow up, with no statistically significant difference (*P* > 0.05).

The SARS-CoV-2 exposure profile and risk of COVID-19 infection among included HCWs who had COVID-19 according to their occupation is described in **Table S5**. The results indicated that the number of COVID-19 cases, despite being higher among HCWs directly exposed to SARS-CoV-2 and at greater risk for COVID-19 infection (physicians, nurses, paramedic nurses), is similar among those HCWs included in BCG revaccinated or unvaccinated groups, regardless of occupation (**Table S5**).

### Safety

The local reactogenicity of the BCG vaccine was evaluated for all groups, revaccinated or unvaccinated. The HCWs revaccinated with BCG reported some vaccine-related AEs. However, as expected, the non-revaccinated group, for which no placebo was used, did not report BCG vaccine-related AEs. Of the total number of individuals (64), sixty-three (98.4%) had local adverse events related to BCG, the three most frequent of which were erythema (n=60; 93.8%), papule (n=50; 78.1%), and crust (n=45; 54.7%). The median number of local AEs related to BCG vaccination was five (IQR=4–6) (**Table 5**). AEs not associated with BCG were reported by 21 HCWs, being more frequent in the revaccinated with BCG group, as reported by 15 HCWs. No participant in the unvaccinated group had severe adverse event (SAE), while three participants in the BCG group (4.7%) had, one of which was related to COVID-19 (**Table 6**) and the other two were not related to BCG vaccination (leg cellulitis and diverticulitis).

**Table 5:** Reactogenicity of the BCG vaccine.

Variables, n (%)^*^	
**Any local AE**	63 (98.4)
**Grade I**	
Erythema	60 (93.8)
Macula	3 (4.7)
Papule	50 (78.1)
Pustule	31 (48.4)
Softening in the center of the lesion	4 (6.3)
Crust	45 (54.7)
Scar	39 (60.9)
Itching	16 (25.0)
Local pain	18 (28.1)
Local heat	6 (9.4)
Local desquamation	4 (6.3)
**Grade II**	
Exudation	3 (4.7)
**Total number of local AE**	308
**Median (IQR)**	5 (4-6)

*Percentages are based on the number of participants in the BCG group. AE, adverse event; IQR, interquartile range.

**Table 6:** Distribution of adverse event during 180 days of follow-up according to the allocation groups†.

Variables	All (n = 131)	Unvaccinated (n = 67)	Revaccinated with BCG (n = 64)	*P -*value^*^
HCWs reporting any AE not related to BCG	21 (16.0)	6 (9.0)	15 (23.4)	**0.03**
Number of AEs	23	4	19	N/A
**Grade I or II AE, n (%)**	20	4	15	0.06
**Grade III AE, n (%)**				
Anemia	1 (0.8)	0	1 (1.6)	0.49
**Severe AE, n (%)**	3 (2.3)	0	3 (4.7)	0.11
Hospitalization due to diverticulitis (CID-10 K57.0)	1 (0.8)	0	1 (1.6)	0.49
Hospitalization due to cellulitis with surgical drainage (CID-10 L03.8)	1 (0.8)	0	1 (1.6)	0.49
Hospitalization due to COVID-19 (CID-10 U07.1)	1 (0.8)	0	1 (1.6)	0.49

^†^Adverse reaction (AE) is defined as any event that was not present before the start of the study (exposure to study vaccination) or any pre-existing event that worsens in intensity and frequency after exposure. Percentages are based on the number of participants in the trial. N/A, not applicable; ^*^Fisher’s exact test.

### Innate Immune Response After BCG Revaccination

To verify the responsiveness of NK IFN-γ- or TNF-α-positive cells, total blood was stimulated with BCG Moscow culture filtrate proteins (CFPs). It was possible to observe that the unvaccinated and revaccinated with BCG groups had similar levels of NK IFN-γ- or TNF-α-positive cells, on both day 1 and day 15 (**Table 7**; *P* > 0.05). The immune response results presented high variability; thus, the fold of increase of these cell populations was evaluated, comparing day 15 relative to day 1 post-randomization. As shown, no differences were observed when the fold of increase of activated cells was evaluated (**Table 7**; *P* > 0.05).

**Table 7:** NK responses to *M. bovis*-CFP stimulation in BCG and unvaccinated groups.

Immune responses^†^	Total (n = 121)	Unvaccinated (n = 61)	Revaccinated with BCG (n = 60)	*P* -value*
**NK IFN - γ^+^ cells**				
Day 1	3.08 (1.70-4.66)	3.08 (1.42-4.60)	3.07 (1.76-5.02)	0.63
Day 15	4.00 (2.48-7.56)	3.87 (2.52-6.59)	4.08 (2.20-8.52)	0.74
**NK TNF - α^+^ cells**				
Day 1	8.14 (2.84-14.35)	7.90 (2.68-13.79)	8.33 (2.83-15.66)	0.60
Day 15	8.50 (2.69-16.97)	9.24 (2.57-17.32)	8.42 (2.80-16.32)	0.90
**IFN fold of increase**	1.48 (0.70-2.93)	1.44 (0.70-3.10)	1.52 (0.51-2.86)	0.83
**TNF fold of increase**	0.94 (0.52-1.86)	0.86 (0.44-1.70)	1.01 (0.60-1.99)	0.26

*Mann-Whitney U test; ^†^Results are presented as median (IQR).

BCG vaccinations were hypothesized as inductors of NK responses that could reduce SARS-COV-2 cell propagation and therefore reduce COVID-19 symptoms. Although no differences were observed in the evaluated immune responses, it was questioned whether previously activated NK cells could have an impact on the symptoms associated with COVID-19. Participants who had a higher fold of increase of NK IFN-γ in the 15–20 days after randomization presented fewer incidences of fever and dyspnea (**Table 8**; *P* < 0.05), indicating a possible role for NK cells that requires further study. No differences were observed when analyzing the other symptoms. Furthermore, the fold of increase analysis of NK TNF-α-positive cells showed no differences between symptomatic and non-symptomatic individuals (**Table 9**; *P* > 0.05).

**Table 8:** Fold of increase of NK IFN- γ^+^ cells after *M. bovis* CFP stimulation according to the different symptoms.

Symptoms^†^	No	Yes	*P -*value*
Cough	1.60 (0.71-3.02)	0.73 (0.43-1.43)	0.06
Fever	1.56 (0.75-3.03)	0.59 (0.52-1.54)	0.02
Sore throat	1.53 (0.70-2.98)	0.44 (0.74-2.05)	0.14
Night sweats	1.46 (0.70-2.88)	N/A	0.45
Dyspnea	1.51 (0.70-2.98)	0.30 (0.2-N/A)	0.05
Anosmia	1.53 (0.70-2.98)	0.70 (0.37-1.69)	0.12
Diarrhea	1.51 (0.71-3.00)	0.58 (0.44-2.33)	0.18
Headache	1.52 (0.70-2.87)	1.32 (066-3.32)	0.86
Runny nose	1.53 (0.71-2.96)	0.77 (0.57-3.64)	0.45
Nasal congestion	1.48 (0.70-2.86)	1.99 (0.57-11.24)	0.48
Ageusia	1.52 (0.71-2.96)	0.59 (0.33-1.99)	0.11
Fatigue	1.46 (0.68-2.87)	1.54 (0.77-3.32)	0.51
Myalgia	1.46 (0.70-2.79)	1.54 (0.58-4.40)	0.63
Arthralgia	1.48 (0.70-2.89)	1.80 (0.28-N/A)	0.76
Nausea	1.46 (0.70-2.95)	N/A	0.51
Vomiting	1.46 (0.70-2.95)	N/A	0.51

*Mann-Whitney U test. ^†^Data are presented as median (IQR); N/A, not applicable.

**Table 9:** Fold of increase of NK TNF-α^+^ cells after *M. bovis* CFP stimulation according to the different symptoms.

Symptoms^†^	No	Yes	*P -*value^*^
Cough	0.98 (0.50-1.90)	0.85 (0.65-1.52)	0.69
Fever	0.99 (0.50-1.89)	0.85 (0.60-1.75)	0.79
Sore throat	0.93 (0.53-1.83)	1.44 (0.42-2.06)	0.53
Night sweats	0.95 (0.53-1.87)	N/A	0.23
Dyspnea	0.94 (0.53-1.88)	0.84 (0.00- N/A)	0.52
Anosmia	0.99 (0.54-1.91)	0.61 (0.21-1.05)	0.16
Diarrhea	0.97 (0.52-1.90)	0.74 (0.44-1.47)	0.55
Headache	0.95 (0.50-1.94)	0.84 (0.66-1.50)	0.93
Runny nose	0.93 (0.47-1.90)	1.41 (0.81-1.63)	0.27
Nasal congestion	0.94 (0.50-1.89)	1.26 (0.67-2.85)	0.56
Ageusia	0.99 (0.53-1.90)	0.85 (0.30-1.26)	0.26
Fatigue	0.95 (0.50-1.89)	0.84 (0.60-1.68)	0.83
Myalgia	0.99 (0.53-1.92)	0.66 (0.28-1.44)	0.36
Arthralgia	0.97 (0.50-1.89)	0.75 (0.66- N/A)	0.61
Nausea	0.95 (0.51-1.87)	N/A	0.94
Vomiting	0.95 (0.51-1.87)	N/A	0.94

^*^Mann-Whitney U test. ^†^Data are presented as median (IQR). N/A, not applicable.

**Figure 3:**
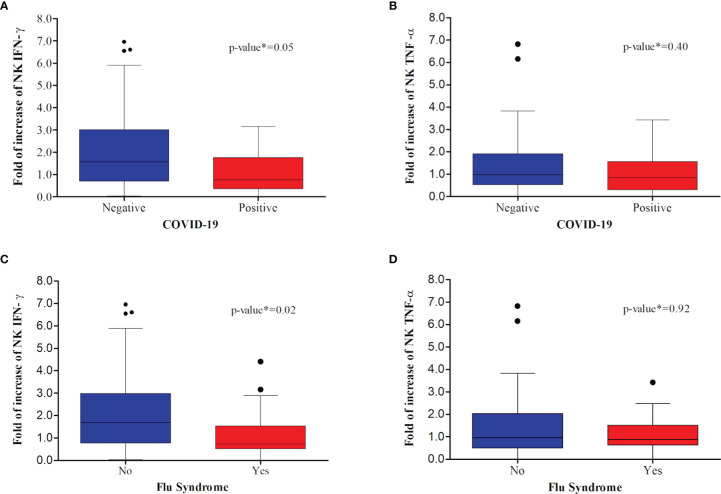
Comparative analysis between confirmed COVID-19 and immunological markers: **(A, C)** fold increase of day 15 relative to day one of NK cells positive for IFN-γ; **(B, D)** fold increase of day 15 relative to day one of NK cells positive for TNF-α. Among individuals that acquired COVID-19 **(A, B)**, there was a significant decrease in the fold increase of NK IFN-γ cells. Similarly, there was a significant decrease in the fold increase of NK IFN-γ cells between those individuals that presented flu syndrome **(C, D)**.

The positivity for COVID-19 or the development of flu-like syndrome symptoms during the trial was analyzed with the fold of increase of NK IFN-γ cells. It was observed that COVID-19-positive individuals or those that presented flu-like symptoms had a lower fold of increase of NK IFN-γ-positive cells when compared to COVID-19-negative individuals or those that presented flu-like syndrome, respectively (**Figures 3A, C**; *P* < 0.05). No differences were observed when the fold of increase of NK TNF-α-positive cells was evaluated (**Figures 3B, D**; *P* > 0.05).

## Discussion

This randomized clinical trial was properly structured to assess the potential of BCG revaccination in protecting against or reducing the severity of COVID-19 among HCWs. The target population were HCWs who, according to the WHO (28), are comprehensively defined as all those engaged in health promotion and maintenance actions, whether in transport, admission, or direct patient care. Here, BCG revaccination has not been shown to be effective in reducing the rate of COVID-19, symptoms or improving innate NK immune response. The study was ended prematurely because a national immunization campaign initiated using specific vaccines for the prevention of COVID-19. One of the campaign’s priority groups was HCWs, discouraging them from participating in this study and, the majority of recruited HCWs had previous COVID-19, one of the study exclusion criteria. As a matter of fact, the national immunization campaign hampered recruitment to achieve the anticipated sample size, introduced a confounding factor and may have limited the findings and conclusions of this study. Nonetheless, to our knowledge, studies using BCG - Moscow strain as well as with HCWs at Brazil had never been analyzed, thus this work contribute to the field and may aid other studies. After the introduction of COVID-19 specific vaccine immunization at Brazil and with the consequent rapid decline of subject’s inclusion in the study, a new theoretical analysis was performed for sample size calculation based on a recent release of COVID-19 incidences among HCWs (26). This assured to ask DSMB to analyze a request for study unmasking, which was consented in August of 2021.

The pandemic is demonstrating that most SARS-CoV-2-infected individuals develop COVID-19 in its asymptomatic form or as a mild form of respiratory infection. However, approximately 20% of cases evolve to its severe form and require specialized care (29). As a result, the rate of hospitalization for complications from COVID-19 has grown exponentially and overloaded health systems. The decision to consider HCWs as a target population was made because the world’s population was isolated in their homes to contain the spread of the SARS-CoV-2 virus, while frontline HCWs were exposed to long and exhausting workdays, often accentuated by insufficient protective equipment, inefficient infection control measures, and accompanying risk of contamination by SARS-CoV-2 (30–35).

In the first half of the pandemic, Gómez-Ochoa et al. (36) demonstrated that 10.1% of COVID-19 cases, registered in 11 countries, involved HCWs. In Brazil, there are no official estimates of the number of HCWs who were contaminated with SARS-CoV-2 or died from complications of COVID-19. However, in public hospitals in Rio de Janeiro, it was reported that 25% of HCWs had COVID-19 in the initial phase of the pandemic, which exceeded the rate recorded by Italy (11%), which was one of the epicenters of COVID-19 in Europe (37, 38). These records worried the entire population, considering that HCWs are essential for the care of patients diagnosed with COVID-19. As a result, HCWs were soon included in the COVID-19 risk group worldwide (32, 39). The HCWs included in this clinical trial were residents of the metropolitan region of Goiânia, which is in the Center-West region of Brazil, where the highest incidence of COVID-19/100 thousand inhabitants was recorded at the time of this writing (40). Most of these HCWs were nurse staff, paramedics (nurses), and medical staff, all of whom were inserted in an environment of high exposure to the SARS-CoV-2 virus, presenting, on average, contact with 20 patients suspected or confirmed for COVID-19 per week. As expected, this group of HCWs, who provided direct patient care, had the highest incidence of COVID-19 during the study (**Table S5** and **Table 2**).

It should be noted that the study did not use a placebo for HCWs allocated to the unvaccinated group because a mock vaccine that provokes similar reactions as the BCG vaccine was not available and, furthermore, the HCWs would easily determine that they were in the placebo group. Other randomized studies have already evaluated the effect of the BCG vaccine in protecting against other diseases without the use of a placebo in the control group. Some of these studies showed results supporting the efficacy and induction of the immune response generated by vaccination with BCG, whereas others did not (7, 41–44). This indicates that the absence of a placebo in the control group does not interfere with the conclusions obtained. However, it should be noted that HCWs not vaccinated with BCG may have been disappointed with the result of randomization and changed their behavior regarding the reporting of health conditions to the telemedicine team, as has already occurred in other studies (45).

On the other hand, once vaccinated with BCG, HCWs were subject to the occurrence of AEs. The reactogenicity provoked by the different strains of BCG is variable and can be influenced by the route of administration (46). The results shown here in this clinical trial revealed that the main reactions reported by HCWs vaccinated with BCG Moscow intradermally were erythema, papule, and crust. These local reactions are similar to those from primary BCG vaccination, as previously described by Hoft et al. (47) during the assessment of clinical reactogenicity of BCG vaccination (Connaught Laboratories, Swiftwater, PA) by the intradermal route. Here, in this clinical trial, AEs were resolved completely and spontaneously over the 180 days of monitoring performed by the telemedicine team, and no SAEs were attributed to BCG vaccination. Considering that approximately a quarter of the world population is infected with latent tuberculosis infection (LTBI) (48), here, in this clinical trial, hypothetical positivity for LTBI did not result in SAEs. In addition, no deaths of BCG-revaccinated or unvaccinated HCWs were reported in this clinical trial, suggesting that BCG vaccination is also not associated with risk of death. This information revealed that the revaccination of adults with BCG Moscow was well tolerated, safe, and, therefore, could be used for new applications in individuals in this age group.

A previous study by Giamarellos-Bourboulis et al. (13) found no difference in the frequency of AEs between the groups vaccinated with BCG and the control in the prevention of infectious conditions in the elderly. Despite this, there is concern regarding the potential harm that could be caused by excessive inflammation induced by the BCG vaccine in patients with COVID-19. Here, in this clinical trial, we observed a higher frequency of AEs not associated with BCG in the revaccinated group. Nonetheless, only one serious event was attributed to COVID-19. In this case, 58 days after inclusion in the clinical trial, one HCW vaccinated with BCG presented dyspnea and hypoxemia, which quickly progressed to respiratory failure unresponsive to non-invasive ventilation, resulting in hospitalization in an ICU and orotracheal intubation. According to information self-reported by the HCW, her clinical condition was characterized by extreme pulmonary involvement, reaching 75% of the organs. After 16 days of hospitalization, this HCW was discharged from the hospital, continued with post-COVID-19 respiratory physiotherapy, and, later, returned to her normal professional activities. The causes that led to the severe prognosis and need for hospitalization have not been elucidated and need further investigation. As BCG revaccination did not induce an increase in the inflammatory innate response, the SAEs observed in this individual could not be attributed to the BCG vaccine. Additionally, here we showed that individuals who developed COVID-19 had a reduced fold of increase in the number of NK IFN-γ cells (**Figure 3**). Despite this SAE, BCG revaccination did not show an increased risk of hospitalization for COVID-19.

In this study, BCG revaccinated individuals presented less COVID-19 cases than control group, although the sample size limited to reach a significant difference. The frequency of symptoms presented by HCWs in the unvaccinated group and those revaccinated with BCG were similar. Notably, this trial was planned in a context without censoring for COVID-19 vaccination. During the study, just after the regulatory agencies’ approval, specific COVID-19 vaccines began to be used for elderly people and HCWs in Brazil (49). Similar findings regarding the efficacy, cumulative incidence rate of COVID-19, and symptom severity were observed even when HCWs were vaccinated against COVID-19 (**Table 4**). Similarly, Amirlak et al. (50) evaluated if BCG revaccination was able to provide COVID-19 protection. Although the study was retrospective in regard to BCG revaccination, a reduction in COVID-19 incidence among the BCG boosted HCWs was observed (50).

A retrospective study carried out by Hamiel et al. (51) compared the incidence of severe COVID-19 in a large cohort of adult Israelis who had and who had not been vaccinated with BCG (unreported strain) in childhood. The results indicated only one case of severe COVID-19 requiring ventilation or admission to the ICU in each group, and no deaths were reported. Similar to our data, the small number of COVID-19 cases in Hamiel et al.’s study could not support a statistically significant difference in the positivity rate for COVID-19, and the number of severe cases was small. Due to these inconclusive results, the authors did not support the idea that childhood BCG vaccination had a protective effect against COVID-19 in adulthood. Despite the similarities between the results, it is prudent to emphasize that Hamiel et al. (51) evaluated the potential of childhood BCG vaccination to protect against COVID-19 in adulthood, rather than recent BCG vaccination.

Interestingly, preliminary data derived from the study BCG-PRIME (NCT04537663) suggested that the BCG vaccine did not protect vulnerable elderly people from COVID-19. Although the study included and monitored more than 6,132 sixty-year-old individuals for 180 days after BCG vaccination, the results revealed that COVID-19 infection in combination with disease symptoms occurred with the same frequency in the elderly from the control group and those vaccinated with BCG. However, it is important to note that almost all individuals participating in the BCG-PRIME study received the BCG vaccine for the first time, as the Netherlands did not adopt a policy of vaccination with BCG in childhood (52).

In contrast, the double-blind, randomized trial (ACTIVATE-2) performed with Greek volunteers demonstrated that BCG revaccination resulted in a 68% risk reduction for total COVID19 diagnoses (OR 0.32. 95% CI 0.13-0.79). However, in that study, the diagnosis of COVID-19 of some participants was based on the clinical diagnosis of possible or probable COVID-19 (26). Another study, carried out with HCWs from Emirates International Hospital (United Arab Emirates) demonstrated potential effectiveness of BCG revaccination in preventing COVID-19 infections (50). Although all HCWs were tested for COVID-19 during the study period, there are two important limitations that should be considered here: the lack of understanding of any confounding factors between the BCG-revaccinated and non-BCG-revaccinated groups that eventually may have influenced the rate of transmission and infection, in addition to the discrepancy between the number of individuals in the two groups (revaccinated with BCG=71 and not revaccinated with BCG=209).

Additionally, study carried out by Rivas et al. (53) demonstrated that HCWs with a history of BCG vaccination presented lower positivity for anti-SARS-CoV-2 IgG or COVID-19 symptoms. Here, it was also observed a reduced number of COVID-19 cases among HCWs revaccinated with BCG, but no significant differences between the groups were observed. Furthermore, it should be noted that the BCG vaccination referred by Rivas et al. (53) is retrospective to the analysis, that is different from a randomized clinical trial.

The reasons why no non-specific effects attributable to BCG revaccination in protecting against or reducing the severity of COVID-19 were observed in the present clinical trial should be investigated by different aspects, including immune response. Many studies are underway to explore the immune response profile of BCG vaccination. Some studies have shown that the revaccination of elderly individuals with BCG Pasteur has contributed to the prevention of acute upper respiratory tract infection (AURTI). This vaccine contributes to an increase in plasma levels of IFN-γ and IL-10 after six months of administration (14). More recently, it was shown that elderly individuals newly vaccinated with BCG Bulgaria had a lower frequency of viral infections during the year. The stimulation of blood mononuclear cells with *Mycobacterium tuberculosis* (Mtb) resulted in an increase in IFN-γ specific to Mtb at 90 days but is not observable at 14 days after vaccination. Stimulation with non-specific components results in the production of IL-6 at 14 and 90 days after vaccination (13). In a study using the BCG SSI vaccine (Danish), it was shown that vaccination appeared to be involved in preventing fungal infections such as Candidiasis. After 14 days of immunization, it was observed that vaccination with BCG induced non-specific pro-inflammatory cytokine-secreting NK cells, such as IL-1β, IL-6, and TNF-α (54). However, in the work evaluated here, no difference in the levels of NK IFN-γ- or TNF-α-positive cells was observed after stimulation with CFPs from *M. bovis* BCG. Some works have shown that after 15–20 days post-BCG vaccination, NK cells are activated, while other studies have shown that this response is maintained for at least three months (11, 54). However, none of these studies used BCG Moscow as a vaccine. Whether the BCG strain may have contributed to the lack of NK responses and consequently to the failure to prevent SARS-CoV-2 infection is still a matter of debate.

It is known that the protection conferred by BCG against tuberculosis depends on the induction of a specific response, Th1 (55); however, this protection differs in terms of the type of strain used worldwide (56). It has been observed that the immune response also presents a different response amplitude, according to the strain used (57). This can be seen in a clinical trial carried out in Uganda (ISRCTN32849447), which tested three different types of strains (BCG Moscow, BCG Bulgaria, and BCG Danish), verifying that both BCG Danish and BCG Bulgaria strains induced IFN-γ and IL-10 in a manner superior to that induced by BCG Moscow (58). When using the BCG vaccine to prevent various infections, such as flu infections, the same effect has been observed. Another clinical trial (ClinicalTrial.gov NCT03296423) used the revaccination of elderly individuals with BCG Bulgaria and demonstrated a reduction in the frequency of viral infections and respiratory infections, which may be related to the cross-effect induced by the BCG vaccine (13). However, in this study, which used the BCG Moscow vaccine, a reduction in the degree of infection by SARS-CoV-2 or in the symptoms presented in individuals who were vaccinated and infected was not observed. The decision to use the BCG Moscow strain in this trial was based on its use in the Brazilian National Immunization Program since 2018. Thus, further studies should be developed to evaluate its protective response against tuberculosis in Brazil.

The protection observed in a work by Giamarellos-Bourboulis et al. (13) has been related to epigenetic modifications in monocytes, which showed increased H3K27 histone acetylation at the *IL-6* and *TNF* genes and increased TNF-α, IL-1β, and IL-10 cytokine synthesis after three months of immunization. Apparently, the BCG Bulgaria vaccine induces an effective heterologous response, which may have contributed to a cross-response against other infections. Different from the results observed by Giamarellos-Bourboulis et al. (13), our work, which used the BCG Moscow strain, evaluated by flow cytometry NK (CD16^+^CD56dim) cells after 14 days of immunization, which did not enhance their production of IFN-γ or TNF-α. Our work, although employing a different methodology, presented similar results as those observed by Kleinnijenhuis et al. (54). In their experiment, cytokines secreted by cultured NK cells stimulated with Mtb antigens after BCG vaccination were evaluated. Kleinnijenhuis and colleagues observed that although other cytokines were increased, neither IFN-γ nor TNF-α were significantly increased upon NK cell stimulation, 14 days after BCG vaccination.

In addition, it has been shown that sex hormones can interfere with the immune response induced by BCG Bulgaria. In the work developed by Koeken et al. (59), referring to 300BCG (NL58553.091.16) and 500FG (NL42561.091.12) studies, it was shown that the response of the BCG Bulgaria vaccine reduced systemic inflammation in vaccinated men. However, the elicited immune response evaluated by the production of IFN-γ by PBMC in response to Mtb antigens was greater in women than in men (59). In this clinical trial, women were overrepresented due to their predominance in the health workforce. Nonetheless, the incidence of COVID-19 was reduced among BCG revaccinated women compared to unvaccinated group, while revaccinated men had a higher risk of developing COVID-19. Unfortunately, due to the limited number of individuals investigated, the differences did not reach statistical significance (**Table S2**). Unfortunately, due to this low number of infected HCWs, we cannot conclude whether sex hormones interfered with the immune response induced by BCG Moscow. Despite the disappointing findings, this clinical trial provided information that should be of interest for the medical and scientific community globally and can inform further studies about how to face future challenges posed by other infectious diseases.

In conclusion, the revaccination of adults with BCG Moscow was safe but did not protect HCWs against COVID-19. BCG Moscow revaccination of HCWs did not induce NK activation.

## Limitation

Caution is required when interpreting the findings presented in this study. One limitation was the fact that many recruited volunteers spontaneously stopped participating for various reasons, but mainly due to previous COVID-19 infection. Furthermore, during the study, just after the regulatory agencies’ approval, specific COVID-19 vaccines began to be used for elderly people and HCWs in Brazil. All HCWs who were being followed and who decided to take a specific vaccine were asked to submit to blood sample collection to assess the possible presence of asymptomatic COVID-19. Even so, HCWs were tested for COVID-19 after 180 days of study enrollment, as provided for in the study design. As the results of this study were objective and were verified through serological and molecular tests or clinical assessment as recommended by the WHO, it is believed that vaccination against COVID-19 did not compromise the analyses. At the time of this trial design, data regarding COVID-19 incidence among HCWs and the population in general were scarce, especially in Brazil, so the reported European incidence rate was used to calculate the sample size. This rate was shown to be higher than that observed among Brazilian HCWs. Nonetheless, the target of 197 participants was the objective of the trial, which was not reached for two main reasons: a high rate of COVID-19 infection among the recruited HCWs, thereby barring inclusion in the study, and the introduction of COVID-19-specific vaccination among HCWs. The reduction of the sample size and the reduced cases of COVID-19 among HCWs hindered the detection of significant differences.

## Data Availability Statement

The raw data supporting the conclusions of this article will be made available by the authors, without undue reservation.

## Ethics Statement

The studies involving human participants were reviewed and approved by Comissão Nacional de ética em Pesquisa -CONEP. The patients/participants provided their written informed consent to participate in this study.

## Author Contributions

APJK was the principal investigator and conceived and designed the trial protocol with help from AK, LRBA, ACC, AROC, MFR, and MBC. LRBA, KMR, RLR, KMCB, ACOC, CCS, RRMF, and CISD performed the recruitment. LA, KR, and CS performed the scheduling for the study participants. APJK, AK, and LRBA ensured the correct storage and use of the BCG vaccine. KMR, KB, ACOC, LSB, CCS, and LRBA assisted with the informed consent collection process and participant inclusion. APJK, ACC, AR, LRBA, and KMR performed the laboratory testing and organized the sample collection. MBC helped in the training of the team involved in conducting the clinical trial. RG was an external member who performed the data curation and statistical analyses. AROC, GS, RLR, RRMF, TV, and MFR performed the clinical follow-up with the study participants for 180 days using telemedicine and/or clinical evaluations. APJK, ACC, and LSB performed the NK experiments and analysis. LRBA, ACC, and RG wrote the original manuscript draft. APJK and AK edited and conducted the final proofreading of the text, and the authors read and approved the manuscript.

## Funding

This trial was funded by Conselho Nacional de Desenvolvimento Científico e Tecnológico/National Council for Scientific and Technological Development (CNPq), Ministério da Ciência, Tecnologia e Inovações (MCTI)/Ministry of Science, Technology and Innovations, Grant numbers: (401206/2020-3; 303671/2019-0; 314366/2020-2). Furthermore, the BCG vaccines were kindly donated by the Brazilian Immunization Program of the Health Ministry of the Brazilian Government/Programa Nacional de Imunização, Ministério da Saúde, Brasil. Neither the funding agency nor the Brazilian Immunization Program had a role in the trial design, the conception, collection, analysis, interpretation, or conclusions, or the decision to write this manuscript. LRBA received fellowship from Coordenação de Pessoal de nível Superior (CAPES-Finance Code 001) and KMR, CCS, ACOC, and AOR received fellowship from CNPq.

## Conflict of Interest

The authors declare that the research was conducted in the absence of any commercial or financial relationships that could be construed as a potential conflict of interest.

## Publisher’s Note

All claims expressed in this article are solely those of the authors and do not necessarily represent those of their affiliated organizations, or those of the publisher, the editors and the reviewers. Any product that may be evaluated in this article, or claim that may be made by its manufacturer, is not guaranteed or endorsed by the publisher.
