# Supplementary material for: Efficacy and Safety of BCG Revaccination With M. bovis BCG Moscow to Prevent COVID-19 Infection in Health Care Workers: A Randomized Phase II Clinical Trial
Source: Front Immunol. 2022 Mar 22;13:841868. doi: 10.3389/fimmu.2022.841868 (PMC8981724; doi:10.3389/fimmu.2022.841868)
Supplement: Supplementary file 1 [file DataSheet_1.pdf]

Supplementary Material

Table S1: Vaccine efficacy based on COVID-19 infection in both scenarios (without censoring for COVID-19 vaccination or with censoring for COVID-19 vaccination) – analysis of 113 individuals with vaccine scar.

**Scenario**	**All**	**Unvaccinated**	**Revaccinated with BCG**
**COVID-19 –** without censoring for COVID-19 vaccination			
Number of COVID-19 cases, n	16	9	7
Cumulative incidence, n/total (%)	16/113 (14.2)	9/57 (15.8)	7/56 (12.5)
Number censored, n (%)	97 (85.8)	48 (84.2)	48 (84.2)
**VE based on HR (95.0% CI)***			24.0 (-109.0 to 72.0)
Person-Years	51.7	25.9	25.8
IR per 100 Person-Years (95.0% CI)	30.8 (17.6-50.1)	34.7 (15.9-66.0)	27.1 (10.9-55.9)
**VE based on IRR (95.0% CI)** †			25.0 (-106.0 to 74.0)
**COVID-19 –** with censoring for COVID-19 vaccination			
Number of COVID-19 cases, n	13	7	6
Cumulative incidence, n/total (%)	13/113 (11.5)	7/57 (12.3)	6/56 (10.7)
Number censored, n (%)	100 (88.5)	50 (87.7)	50 (89.3)
**VE based on HR (95.0% CI)***			20.0 (-148.0 to 74.0)
Person-Years	36.5	18.0	18.5
IR per 100 Person-Years (95.0% CI)	35.6 (19.0-60.9)	38.9 (15.6-80.1)	32.4 (11.9-70.6)
**VE based on IRR (95.0% CI)**†			25.0 (-134.0 to 77.0)

Note: The primary endpoint was COVID-19 defined as the presence of positivity by RT-PCR or IGM or IgG serology for COVID-19 defined as presented in methods. IRR = incidence rate ratio; HR=hazard ratio; VE = vaccine efficacy; CI = confidence interval. * Vaccine efficacy based on 1-HR obtained in Cox proportional model adjusted for age and sex; † Vaccine efficacy based on 1-IRR obtained in Poisson model adjusted for age and sex, with the natural logarithm (log[n]) of time at risk as offset variable.

Table S2. Vaccine efficacy based on COVID-19 infection by sex without censoring for COVID-19 vaccination.

	Male			Female		
	**All**	**Unvaccinated**	**BCG revaccination**	**All**	**Unvaccinated**	**BCG revaccination**
Number of COVID-19 cases, n	5	1	4	14	10	4
Cumulative incidence, n/total (%)	5/31 (16.1)	1/11 (9.1)	4/20 (20.0)	14/100 (14.0)	10/56 (17.9)	4/44 (9.1)
Number censored, n (%)	26 (83.9)	10 (90.9)	16 (80.0)	86 (86.0)	46 (82.1)	40 (90.9)
**VE based on HR (95.0% CI)***			-132.0 (-2,040.0-75.0)			52.0 (-52.0-85.0)
**VE based on IRR (95.0% CI) †**			-123.0 (-4,350.0-69.4)			52.0 (-42.0; 87.0)

Note: The primary endpoint was COVID-19 defined as the presence of positivity by RT-PCR or IGM or IgG serology for COVID-19 defined as presented in methods. IRR = incidence rate ratio; HR=hazard ratio; VE = vaccine efficacy; CI = confidence interval. * Vaccine efficacy based on 1-HR obtained in Cox proportional model adjusted for age; † Vaccine efficacy based on 1-IRR obtained in Poisson model adjusted for age, with the natural logarithm (log[n]) of time at risk as offset variable.

Table S3: Comparison of symptoms between vaccinated with BCG and unvaccinated groups for positive COVID-19 cases

Signs and symptoms	Total (n=19) †	Unvaccinated (n = 11) †	Revaccinated with BCG (n = 8) †	p-value*
Systemic	14 (73.7)	8 (72.7)	6 (75.0)	1.00
Night sweats	1 (5.3)	1 (9.1)	0	1.00
Fever	6 (31.6)	2 (18.2)	4 (50.0)	0.32
Myalgia	6 (31.6)	4 (36.4)	2 (25.0)	1.00
Fatigue	8 (42.1)	5 (45.5)	3 (37.5)	1.00
Arthralgia	1 (5.3)	0	1 (12.5)	0.42
High respiratory	10 (52.6)	7 (63.6)	3 (37.5)	0.37
Sore throat	3 (15.8)	3 (27.3)	0	0.23
Nasal congestion	3 (15.8)	3 (27.5)	0	0.23
Runny nose	6 (31.6)	3 (27.3)	3 (37.5)	1.00
Low respiratory	9 (47.4)	5 (45.5)	4 (50.0)	1.00
Cough	7 (36.8)	5 (45.5)	2 (25.0)	0.63
Dyspnea	3 (15.3)	1 (9.1)	2 (25.0)	0.55
Gastrointestinal	3 (15.8)	2 (18.2)	1 (12.5)	1.00
Diarrhea	5 (3.8)	2 (3.0)	3 (4.7)	0.68
Nausea	0	0	0	N/A
Vomiting	0	0	0	N/A
Neurological	13 (68.4)	7 (63.6)	6 (75.0)	1.00
Anosmia	7 (36.8)	5 (45.5)	2 (25.0)	0.63
Ageusia	8 (42.1)	5 (45.5)	3 (37.5)	1.00
Headache	10 (52.6)	6 (54.5)	4 (50.0)	1.00
Flu syndrome ‡	14 (73.7)	8 (72.7)	6 (75.0)	1.00

N/A = Not applicable; * Fisher's exact Test; † Data are presented as n (%). ‡ Participants that had two or more symptoms related to Flu syndrome.

Table S4. Comparison of symptoms between BCG and control groups

Signs and symptoms	Total (n=131) †	Unvaccinated (n=67) †	Revaccinated with BCG (n=64) †	p-value*
Headache	21 (16.0)	9 (13.4)	12 (18.8)	0.41
Cough	16 (12.2)	8 (11.9)	8 (12.5)	0.92
Runny nose	15 (11.5)	8 (11.9)	7 (10.9)	0.86
Fatigue	14 (10.8)	6 (9.1)	8 (12.5)	0.53
Fever	13 (9.9)	4 (6.0)	9 (14.1)	0.12
Myalgia	12 (9.2)	5 (7.5)	7 (10.9)	0.49
Sore throat	11 (8.4)	7 (10.4)	4 (6.3)	0.39
Anosmia	8 (6.1)	5 (7.5)	3 (4.7)	0.72
Ageusia	8 (6.1)	5 (7.5)	3 (4.7)	0.72
Diarrhea	5 (3.8)	2 (3.0)	3 (4.7)	0.68
Dyspnea	3 (2.3)	1 (1.5)	2 (3.1)	0.61
Arthralgia	2 (1.5)	0	2 (3.1)	0.24
Nasal congestion	5 (4.6)	5 (7.5)	1 (1.6)	0.21
Night sweats	1 (0.8)	1 (1.5)	0	1.00
Nausea	1 (0.8)	0	1 (1.6)	0.49
Vomiting	1 (0.8)	0	1 (1.6)	0.49
Flu syndrome‡	28 (21.4)	13 (19.4)	15 (23.4)	0.57

* Pearson's chi-squared test or Fisher's exact test. † Data are presented as n (%); ‡ Participants that had two or more symptoms related to Flu syndrome.

Table S5. Number of COVID-19 cases according to occupation and allocated group (unvaccinated and BCG)

Occupation	All	Unvaccinated	Revaccinated with BCG
Administrative staff	2/13 (15.4)	2/4 (50.0)	0/9 (0.0)
Nurse staff	8/48 (16.7)	4/27 (14.8)	4/21 (19.0)
Dental professionals	0/9 (0.0)	0/3 (0.0)	0/6 (0.0)
Laboratory staff	0/12 (0.0)	0/7 (0.0)	0/5 (0.0)
Nutritionist	0/4 (0.0)	0/1 (0.0)	0/3 (0.0)
Community health agents	1/6 (16.7)	1/5 (20.0)	0/1 (0.0)
Paramedics	3/18 (16.7)	1/9 (11.1)	2/9 (22.2)
Physicians	4/10 (40.0)	2/6 (33.3)	2/4 (50.0)
Other health care workers professionals	1/11 (9.1)	1/5 (20.0)	0/6 (0.0)
All	19/131 (14.5)	11/67 (16.4)	8/64 (12.5)

Data are presented as number of COVID-19 cases / number of participants in each occupation (accumulated incidence in %).
